# Extrahepatic factors in hepatic immune regulation

**DOI:** 10.3389/fimmu.2022.941721

**Published:** 2022-08-16

**Authors:** Shaoying Zhang, Shemin Lu, Zongfang Li

**Affiliations:** ^1^ National-Local Joint Engineering Research Center of Biodiagnosis & Biotherapy, The Second Affiliated Hospital, Xi’an Jiaotong University, Xi’an, China; ^2^ Shaanxi Provincial Clinical Medical Research Center for Liver and Spleen Diseases, The Second Affiliated Hospital, Xi’an Jiaotong University, Xi’an, China; ^3^ Shaanxi International Cooperation Base for Inflammation and Immunity, The Second Affiliated Hospital, Xi’an Jiaotong University, Xi’an, China; ^4^ Key Laboratory of Environment and Genes Related to Diseases, Ministry of Education, Xi’an, China

**Keywords:** hepatic immune regulation, extrahepatic factor, liver disease, immune cells, inflammation

## Abstract

The liver is a site of complex immune activity. The hepatic immune system tolerates harmless immunogenic loads in homeostasis status, shelters liver function, while maintaining vigilance against possible infectious agents or tissue damage and providing immune surveillance at the same time. Activation of the hepatic immunity is initiated by a diverse repertoire of hepatic resident immune cells as well as non-hematopoietic cells, which can sense “danger signals” and trigger robust immune response. Factors that mediate the regulation of hepatic immunity are elicited not only in liver, but also in other organs, given the dual blood supply of the liver *via* both portal vein blood and arterial blood. Emerging evidence indicates that inter-organ crosstalk between the liver and other organs such as spleen, gut, lung, adipose tissue, and brain is involved in the pathogenesis of liver diseases. In this review, we present the features of hepatic immune regulation, with particular attention to the correlation with factors from extrahepatic organ. We describe the mechanisms by which other organs establish an immune association with the liver and then modulate the hepatic immune response. We discuss their roles and distinct mechanisms in liver homeostasis and pathological conditions from the cellular and molecular perspective, highlighting their potential for liver disease intervention. Moreover, we review the available animal models and methods for revealing the regulatory mechanisms of these extrahepatic factors. With the increasing understanding of the mechanisms by which extrahepatic factors regulate liver immunity, we believe that this will provide promising targets for liver disease therapy.

## Introduction

The liver is the largest organ engaged in metabolic, nutrient storage and detoxification activities, but has increasingly been recognized as a unique immune organ with its own immune features. Since the dual blood supply of portal venous and systemic blood transport a large number of foreign but harmless molecules, the liver immune cells are largely in an activated state due to continuous exposure to low concentrations of antigens, and the default immune status of the liver is anti-inflammatory or immune-tolerant (1–4). Generally, the hepatic immune system tolerates harmless molecules under healthy condition. In face of immune activation challenge posed by pathogens or tissue damage, however, the liver could mount rapid and robust immune response and attempt to resolve inflammation to maintain liver homeostasis. Otherwise, failure to clear ‘dangerous’ stimuli or appropriately regulate activated immune mechanisms can lead to chronic and pathological inflammation (5–8). Maintenance of liver function requires a balance between immunity and tolerance, therefore, appropriate regulation of the complex hepatic immune activities is necessary.

Chronic liver disease is generally a multi-stage, multi-hit process; it is therefore not surprising that multiple cells within the liver contribute to the immune regulation during disease progression. Active modulation of immune responses in the liver could stem from its unique microenvironment including certain cell types like resident immune cells (9–13), hepatocytes (14), hepatic stellate cells (HSCs) (15) and liver sinusoidal endothelial cells (LSECs) (16, 17). In addition to complications relating to the liver, patients with chronic liver disease also develop concomitant extrahepatic functional disturbances of multiple organ systems (18–20), which in turn affect the progression of liver disease. Given this, the contribution of signals outside the liver to the ‘balance control’ of hepatic immune calls for meticulous exploration. In this review, we briefly describe the changes in hepatic immune status from homeostasis to disease, as well as the cellular, molecular and neural factors that mediate these changes. From the perspective of organ-organ communication, we elaborate on the effects of other organs on hepatic immune regulation and liver disease progression, and discuss the available animal models and methods for revealing the regulatory mechanisms of the extrahepatic factors.

## Hepatic immunity and its regulation

The liver has its special intraparenchymal vascular conduits named hepatic sinusoid. The hepatic sinusoids involve multiple and disparate cell types: LSECs form the walls, HSCs harbor in the Space of Disse between the sinusoidal wall and the adjacent hepatocytes, and various immune cells locate within the sinusoid (21). All these cells play active roles in regulating the hepatic immune. The liver is considered a unique immunological organ for its predominant innate immune role, as it contains an unusually large number of innate immune cells, including myeloid cells like macrophages, dendritic cells (DCs), innate lymphocytes like natural killer (NK) cells, innate lymphoid cells (ILCs), and innate-like T lymphocytes like NKT cells, and γδ T cells (22). Of course, the adaptive immune cells (T and B cells) also count in the hepatic immunity (23). Here, we outline the hepatic immune state in homeostasis and their alterations upon activation, and summarize the mechanisms underlying the alterations.

### Orchestrated tolerant hepatic homeostasis during healthy state

Hepatic resident immune cells together with non-hematopoietic cell populations maintain hepatic immune tolerance during healthy state. The liver resident macrophages, Kupffer cells (KCs), play a key role by producing anti-inflammatory mediators such as IL-10 and prostaglandins (24, 25), and downregulating expression of co-stimulatory molecules to limit the adaptive immune response (26). Hepatic resident DCs appear phenotypically immature and are less potent activators of T cells, and shown to produce significantly more IL-10 compared with peripheral derived myeloid DCs (27). Non-hematopoietic cells such as LSECs, HSCs and hepatocytes possess the ability to directly present antigen to T cells, but their presentation of antigens in the liver biases T cells towards tolerance for their lack of co-stimulatory molecules (e.g., driving naïve CD4^+^ T cells differentiation to regulatory T cells rather than to T helper cells) (28–31). Meanwhile, LSECs and hepatocytes constitutively express IL-10 and TGF-β (32, 33). Besides, the healthy liver also has basal expression of pro-inflammatory cytokines (including IL-2, IL-7, IL-12, IL-15 and IFN-γ). The complex cytokine milieu helps orchestrate the homeostasis (34).

### Activated hepatic immunity upon challenge

Once the hepatic homeostasis sheltered by local and systemic tolerance is disrupted, the innate immune system is first activated, driving the full development of inflammatory hepatocellular injury. Depending on the underlying liver disease, such as viral hepatitis, autoimmune hepatitis, cholestasis, liver ischemia reperfusion or metabolic associated steatohepatitis, various triggers mediate immune-cell activation. The initiative inflammatory activation of HSCs and KCs results in the chemokine-mediated infiltration of monocytes (35), neutrophils (36), NK and NKT cells (37). KCs and the recruited monocyte-derived macrophages (MoMFs), as key cellular components of the liver, adapt their phenotype to local signaling, taking an active part in either inflammation or the subsequent resolution (38, 39). The ultimate outcome of the intrahepatic immune response depends largely on the functional diversity of macrophages. Innate lymphocytes like NK and NKT cells are another source of immune-regulatory cytokines in diseased livers, contributing to the elimination of activated myofibroblasts and infected or injured cells (40). These disease conditions are also closely linked to T cell immunity, in which the heterogeneous pool of hepatic T cells is activated upon shifting to inflammation (41, 42), Th1 and Th17 responses are induced, resulting in the secretion of immune-stimulatory cytokine as well as direct cytopathic function (43). A systematic understanding of the initiation and regulation of the activated hepatic immune regulation is critical for the development of liver disease therapy strategies based on intervention of hepatic immunity.

### Hepatic immune regulation

Mechanisms of hepatic immune regulation involves cell activation, molecule interaction and neural signal transmission. At the cellular level, most types of liver ‘insults’ damage epithelial cells, leading to the release of inflammatory mediators and the initiation of inflammatory cascade. In response to these inflammatory signals, KCs are first activated (44), releasing pro-inflammatory mediators that lead to the recruitment of circulatory-derived immune cells into the inflamed liver (45). MoMFs, as the primary leukocytes being recruited, could further recruit T cells and neutrophils to promote fibrosis by generating pro-inflammatory cytokines (e.g., TNF-α, IL-6, and IL-1β), and secreting pro-inflammatory chemokines (e.g., CCL2, CCL5, and CXCL2), thus further amplifying the inflammatory response (46–48). Also, the behavior of MoMFs themselves is regulated by the complex hepatic microenvironment. Emerging understandings of hepatic macrophage heterogeneity identify a group of CD11b^hi^F4/80^int^LY6C^low^ restorative macrophage as a phenotypical switch subpopulation derived from the pro-inflammatory LY6C^hi^ subset, contributing to inflammation resolution (49). Another important myeloid cells, DCs, exert their role in hepatic immune regulation by forming a bridge between the innate and the adaptive immune system (50). Although T cells are also activated during hepatic inflammation, the mechanisms of their activation and “shift” (to Th1 and Th17) are not fully elucidated, which might require further analysis of the interaction between hepatic DCs and T cells.

Molecularly, pathogen-associated molecular patterns (PAMPs) and danger/death-associated molecular patterns (DAMPs) are the most famous “danger signals”. PAMPs are conserved structures vital to pathogens, presenting in microbes and absent in eukaryotes (51, 52). DAMPs represent damaged cells of the host which are a threat to self (53). PAMPs and DAMPs initiate the immune response *via* pattern recognition receptors (PRRs), which are present in immune cells, as well as in LSECs and HSCs (51, 54, 55). The recognition of pathogen molecules by PRRs would lead to activation of the complement cascade, cytokines, antimicrobial peptides and antigen-presenting cells, resulting in a complex interplay of pro- and anti-inflammatory responses and immunogenic and suppressive responses in the host (53, 56). Among PRRs, Toll-like receptors (TLRs) are most extensively studied, responding to most DAMPs and PAMPs, and have a major influence in liver diseases (57). Nucleotide binding oligomerization domain-like receptors (NLRs) represent another subtype of PRRs, for example, NLPR3 is one of the NLRs expressed on inflammasome (44). Inflammasomes are intracellular multiprotein complexes that sense intracellular danger signals including DAMPs, PAMPs, and ROS, and the activation of inflammasomes triggers a pro-inflammatory response commonly associated with caspase-1 activation followed by activated secretion of IL-1β and IL-18 (58). In addition to serving as an important mechanism for macrophage activation signal transduction, inflammasome hyperactivation can also result in hepatocyte pyroptosis, a specific form of cell death, leading to increased liver inflammation and fibrosis development in mice (59). Besides, exosomes have also been identified to play important roles in hepatic immune regulation by mediating the intrahepatic cell-cell communication and transmission of information from other organs to the liver (60).

Furthermore, the liver is innervated by both the sympathetic and the parasympathetic nerve systems. These nerves are derived from the splanchnic and vagal nerves that surround the portal vein, hepatic artery, and bile duct. The nervous system and the immune system communicate in response to pathogen invasion, tissue injury, and other “insults” (61). Intrahepatic efferent nerves endings containing catecholaminergic (releasing neurotransmitters like epinephrine and norepinephrine), cholinergic (releasing neurotransmitter acetylcholine) nerves terminate at the Space of Disse (62, 63), and trigger responsiveness in effector cells. Findings in preclinical conditions have indicated the use of vagus nerve stimulation and acetylcholine (Ach) receptor agonists and centrally acting AchE inhibitors as therapies for liver inflammatory diseases. The exact mechanisms of hepatic nervous system regulating the function of hepatic immune cells and secretion of effector factors remain to be further studied.

## Extrahepatic factors and hepatic immune regulation

**Figure 1:**
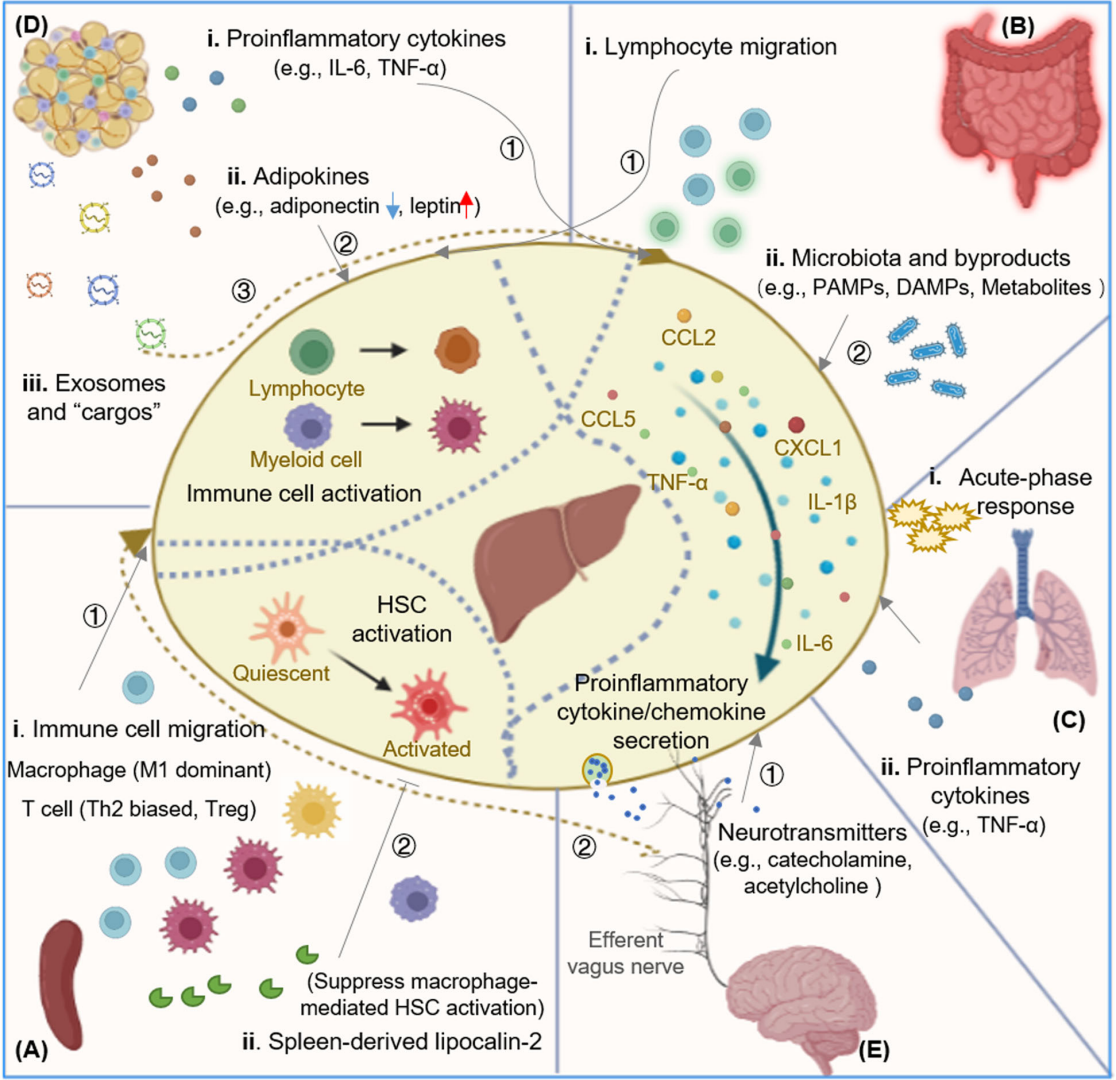
Mechanisms of extrahepatic factors regulating hepatic immunity. Extrahepatic factors in hepatic immune regulation. **(A)** Spleen and hepatic immune regulation. The spleen exerts its role in hepatic immune regulation by affecting the composition of both innate and adaptive immune cells. Spleen-derived Lcn-2 suppresses macrophage­ mediated HSC activation. **(B)** Gut and hepatic immune regulation. Intestinal microbiota and their byproducts (including PAMPs, DAPMs, and metabolites) could be translocated to the liver to active hepatic immune cells and promote the secretion of pro-inflammatory cytokines. Active lymphocytes could also be recruited from the gut into liver to modulate hepatic immunity. **(C)** Lung and hepatic immune regulation. Hepatic acute phase response is induced by the crosstalk between lung and liver communication, and pro-inflammatory cytokine like TNF-a acts as "shuttle" factor in modulating the lung-liver axis. **(D)** Adipose tissue and hepatic immune regulation. Adipose tissue-derived pro­inflammatory cytokines, adipokines, and exosomes modulate hepatic immunity by activating hepatic immune cells and promoting the secretion of pro-inflammatory cytokines. Adipose tissue macrophages are activated and migrate to the diseased liver. **(E)** Brain and hepatic immune regulation. The hepatic inflammatory signals are transmitted and integrated in the CNS, and then descend *via* sympathetic and efferent vagus nerve fibers, releasing catecholamine and acetylcholine through hepatic nerve endings and modulating the liver immune response. Lcn-2, lipocalin-2; HSC, hepatic stellate cell; PAMP, pathogen­ associated molecular pattern; DAMP, danger/death-associated molecular pattern; CNS, central nervous system; KC, Kupffer cell; NKT, natural killer T cell.

Hepatic immunity is a complex and adaptable process, which is flexibly regulated by the changing hepatic responses in homeostasis and various disease states. In addition to complications relating to the liver, patients with liver disease often develop concomitant extrahepatic functional disturbances of multiple organ systems; and the liver itself also intensively participates in the acute phase reaction in response to inflammation that occurs in other organs. There have been clues of the liver communicating with other organs through molecular and cellular mediators and the nervous system, but how these extrahepatic factors are involved in hepatic immune regulation is incompletely understood. Below, we discuss in detail the various ways in which other organs regulate hepatic immunity (**Figure 1**).

### Spleen and hepatic immune regulation

The spleen is the largest immune organ and plays a critical role in the production of various immune cells and numerous cytokines. Compared with other tissues and organs, the spleen is more closely linked with the liver in anatomical structure and function, all spleen blood flows into liver through the portal vein, which facilitates transportation of immune mediators such as immune cells and cytokines into the liver. In chronic liver diseases, splenomegaly and hypersplenism are always manifested following the development of portal hypertension. Splenectomy has been reported to have a role of ameliorating patients’ condition or suppressing liver fibrosis in clinical condition (64–66). Moreover, a large number of studies have shown that the abnormal spleen is involved in the modulation of hepatic immunity, and the role of the so-called “liver-spleen axis” is gaining increasing attention in liver diseases (67–69).

#### Spleen-mediated hepatic immune cell alteration

The spleen exerts its role in hepatic immune regulation by affecting the composition of both innate and adaptive immune cells in liver (**Table 1**). For innate immune cells, the spleen serves as a reservoir of monocytes, contributing to the heterogeneity of hepatic macrophages which are indispensable for rapid responses to liver injury (81). In mice with diet-induced NAFLD, macrophages produced increased inflammatory cytokines like TNF-α and IL-6 (M1-type) were increased, but macrophages mainly secreting the anti-inflammatory IL-10 were unchanged (70, 82). The increased hepatic macrophages during the progression of NAFLD was indicated to migrate from BM to liver *via* the spleen (70). The similar M1 dominant phenotype was also observed in a CCl_4_-induced rat liver fibrosis model. In this model, the increased monocyte recruitment and the establishment of an M1-dominant hepatic macrophage phenotype was facilitated by up-regulated secretion of hepatic CCL2, which was prompted by splenic macrophages (71). In another TAA-induced liver fibrosis model, splenectomy attenuated murine liver fibrosis with hypersplenism and stimulated accumulation of Ly-6C^lo^ macrophages in the liver (83). Our previous study in a chronic restraint stress prompted hepatocellular carcinoma mice model also found that splenectomy could inhibit tumor growth and prevent the increase of macrophage in tumor tissues (72). Clinically, patients suffered with liver cancer showed improved prognosis upon splenectomy, but this is only viable in the subgroup with an increased neutrophil-lymphocyte ratio (NLR) and increased infiltration of CD163^+^ tumor associated macrophages (TAMs) in the tumor stroma (84), indicating the crucial role of spleen-derived macrophages in tumor progression.

**Table 1:** Immune cells migrating from extrahepatic organs to the liver.

Cell types	Cell source	Cell feature and fonction	Liver disease model	Reference
Macrophage	Spleen	CD68+F4/8ü+	Mice/ Diet-induced NAFLD	(70)
		Produce more TNF-a and IL-6		
Macrophage	Spleen	Promote CCL2 secretion by hepatic Mcp Establish an Ml-dominant hepatic Mcp phenotype	Rat/ CC14-induced liver fibrosis	(71)
Myeloid cells	Spleen	Promote hepatocellular carcinoma growth	Mice/ Hepatocellular carcinoma	(72)
			under chronic restraint stress	
T cell	Spleen	Transform the cytokine balance into Th2 dominance	Mice/ CC14-induced liver fibrosis	(73)
		Bias the hepatic T cells toward Th2		
T cell	Spleen	CXCR3+ Tregs account for a considerable	Mice, human/ Liver fibrosis	(74, 75)
		proportion	caused by Schistosoma	
		Modify T helper cytokine balance	japonicum infection	
Lymphocyte	Gut	CCR9+	Human/ Inflammatory bowel	(76, 77)
		Drive hepatobiliary destruction in PSC	disease	
B cell	Gut	Reactive to commensal bacteria	Mice, human/ Alcoholic liver	(78)
		Clear gut-derived antigens	disease	
		Protect organs from pathogens		
ATM	Adipose	Promote insulin resistance and inflammatory	Obese mice/ High-fat diet	(79, 80)
	tissue	response		

Mcp, macrophage; Th2, T helper 2; PSC, primary sclerosing cholangitis; ATM, adipose tissue macrophage.

As for hepatic adaptive immune cells, their composition can also be altered by the spleen. In a CCl_4_-induced liver fibrosis mice model, researchers found that splenectomy biased the Th1/Th2 balance in the liver towards Th1 dominance. Upon the transplantation of labelled splenocytes into the spleens of syngeneic wild-type mice, labelled CD4^+^ lymphocytes appeared in the liver after fibrosis induction, among which the vast majority were Th2 lymphocytes (73). That is, Th2-dominant splenic lymphocytes were recruited to the liver and promoted liver fibrosis by transforming the cytokine balance into Th2 dominance, and splenectomy suppressed the progression of fibrosis at least partly by restoring the Th1/Th2 balance. In the Schistosoma japonicum infection caused liver fibrosis model, dynamic changes of lymphocyte populations in the spleen and concurrent upregulation of chemokines and cell adhesion molecules in the liver also suggested a recruitment of active immune cells from spleen to the liver (74), among which CXCR3^+^ Tregs were supposed to occupy a considerable proportion of the lymphocytes that migrate from spleen to Th1-infiltrated liver tissues to regulate liver fibrosis (75).

To date, the role of spleen in affecting the composition of hepatic immune cell composition have been well acknowledged, however, these conclusions are mostly based on splenectomy. We have limited information about whether splenic immune cells are directly recruited to the liver, and whether the spleen delivers specific subtypes of immune cells to liver at different stages of liver disease progression. In addition, it is also worth investigating whether and how the diseased liver regulates the composition of splenic immune cells. A better understanding of these issues is crucial to delineate spleen-mediated hepatic immune regulation and lay a foundation for developing novel strategies for liver disease immunotherapy.

#### Spleen-derived factors mediated hepatic immune regulation

Lipocalin-2 (Lcn2) is an antimicrobial protein that regulates macrophage activation. Significant increase of splenic Lcn2 was detected in mice with liver fibrosis, but levels of all other measured cytokines were unchanged. The splenectomy mice showed enhanced liver fibrosis and inflammation, accompanying significantly decreased Lcn2 in portal vein. Upon treatment with recombinant Lcn2 *in vitro*, LPS-stimulated primary KCs produced less TNF-α and CCL2, and the activation of HSCs was suppressed by co-culture with rLcn2-treated KCs. The mechanism of splenic protection against liver fibrosis development may involve the splenic Lcn2. The splenic Lcn2 might have an important role in regulating hepatic immune tolerance during the development of liver fibrosis (85). The liver has an extraordinary capacity to regenerate upon various injuries (86). Several experimental studies have demonstrated that removal of the spleen accelerates liver regeneration and inhibits the development of liver fibrosis  (71, 87–89), indicating a certain role of the spleen in liver regeneration. TGF-β is recognized as the critical factor in the performance of spleen to inhibit liver regeneration in both the thioacetamide-induced liver fibrosis rat model (90) and the partial hepatectomy rat model (91). Upon injury, the liver goes through a process from initiation and proliferation to resolution and repair. These results suggest that the spleen might also plays a role in the resolution and repair of fibrotic liver.

### Gut and hepatic immune regulation

Close anatomical and physiologic connections exist between the gut and liver. These two organs are linked through the portal circulation, and the liver receives 70% of its blood supply from the intestine through the portal vein. Therefore, the liver acts as the first line of defense against gut-derived antigens, and one of the most exposed organs to gut-derived toxic factors, such as bacteria and bacterial byproducts (92). Besides, the gut and liver also communicate through biliary tract and systemic circulation, the bidirectional crosstalk facilitates the formation of gut-liver axis.

The gut microbiota consists of various microorganisms that normally coexist in the gut and have a role of maintaining the homeostasis of the host. A shift in gut microbiota composition can lead to activation of the mucosal immune response, causing homeostasis imbalance. This imbalance results in the translocation of metabolites and components derived from the gut microbiota, and also leads to the transport of active immune cells to the liver, thus inducing pathologic effects in the liver (8, 93). Clinical observations and animal experimental studies reveal that the gut barrier damage seldom leads to liver injury independently but aggravates pre-existing liver diseases, and the circulatory homeostasis is largely intact in patients with early cirrhosis and portal hypertension (94). With the progression of liver fibrosis, regardless of the cause, pathophysiology extends to the intestinal tract with increased intestinal permeability and overgrowth of gut microbiota. The microbiota and their byproducts could then enter the liver through the portal vein, causing inflammation and damage in the liver (95–100). Extra evidence of this process is provided by transplantation of intestinal microbiota from humans with acute alcoholic hepatitis into germ-free and conventionally housed mice (101, 102). Intestinal microbiota entering the liver regulates hepatic immunity *via* several mechanisms.

#### Intestinal microbiota and their byproducts activate hepatic immune cell response

The signature and role of gut microbiota in different liver diseases has been reviewed elsewhere (103). Here we emphasize its role in hepatic immune regulation and attempt to disclose the mechanism of its influence on liver disease progression from the perspective of immunity.

In a ConA-induced hepatitis model, ConA treatment failed to activate hepatic NKT cells in germ-free mice, but supplementation with killed intestinal bacteria facilitate NKT cell activation (104). Also, another study with mice transplanted with gut microbiota from a patient with severe ALD found that the mice developed more severe liver inflammation with increased NKT cells (101). Growing evidence suggests that γδ T cells expand in response to invading bacterial pathogens and modulate tissue injuries (105, 106). As the major producers of IL-17A, the production of IL-17A by hepatic γδ T cells was found modulated by the commensal bacterial load (107). Both NKT and γδ T cells are innate lymphocytes enriched in the liver (108). That is, gut-derived microbiota activate innate lymphocytes in the liver, although the mechanism is unclear.

PAMPs, conserved structures vital to microbiota, are one of the main mechanisms of microbiota to activate hepatic immunity. Once gut-derived PAMPs enter the liver through the portal vein, they can activate cells expressing PRRs (e.g., TLRs, NLRs) and induce inflammation (109). Examples of relevant gut-derived PAMPs include LPS, lipoteichoic acid (LTA), and β-glucan (110). LPS is one of the most well-known components of gram‐negative bacteria, and activates hepatic macrophages through interaction with TLR4 (103, 111). Indeed, in NAFLD patients, LPS-induced activation of liver macrophages is associated with inflammation and fibrosis, TLR4 knockout attenuates experimental NASH (112). LTA is a gram-positive microbial component, functions through up-regulating the expression of cyclooxygenase-2 (COX-2), and COX-2-mediated prostaglandin E2 (PGE2) production suppresses antitumor immunity, thereby contributing to the immunosuppressed hepatic microenvironment (93). 1,3-β-glucan, from the overgrowth of fungi, on the one hand binds to the C-type lectin domain family 7 member A (CLEC7A) of KCs and possibly other bone marrow–derived cells and promotes liver inflammation, on the another hand increases PGE2 production in the liver (113, 114). DAMPs, another famous “danger signal”, have also been identified deriving from the intestine and triggering immune response in the liver. In the ASC−/- mice on a high-fat diet (HFD), Chen and colleagues (115) identified a DAMP molecule high-mobility group protein B1 (HMGB1) as a “cargo” transported by exosomes from the intestine to the liver, triggering hepatic steatosis. Recently, injection of intestinal exosomes from ischemia/reperfusion (I/R) mice to healthy mice was also shown able to cause macrophage infiltration, M1 polarization, and liver inflammation in mice (116).

In addition, metabolites derived from the gut microbiota also play roles in hepatic immune regulation. BAs (bile acids) represent one of the classic components that function in the gut-liver axis. BAs including chenodeoxycholic acid and deoxycholic acid (DCA), could upregulate NLRP3 in hepatic macrophages, contributing to cholestatic liver diseases (117). Another important component, D-lactate, could protect against pathogen dissemination by upregulating the phagocytic capability of KCs, thereby generating an intravascular immune firewall (118).

#### Intestinal microbiota and their byproducts shape hepatic immune milieu

The gut microbiota also shape the hepatic immune milieu by regulating inflammatory cytokines (119–121). In the alcohol-related liver disease (ALD) model, LPS-TLR4 signal in macrophages was delivered by the recruitment of adapter molecules, such as MyD88 and TRIF (122). MyD88-mediated NF-κB activation produced pro-inflammatory cytokines (e.g., TNF-α, IL-6, and IL-1β) and chemokine CCL2, whereas the TRIF pathway induced the production of type-I interferons (123, 124). In murine liver fibrosis, translocation of gut microbiota induced tonic type I IFN expression in the liver, and then conditioned liver myeloid cells to produce high concentrations of IFN in response to intracellular infection with bacteria. Such IFN-receptor signaling also caused myeloid cell IL-10 production that corrupted antibacterial immunity, leading to loss of infection control and to infection-associated mortality (125). The prominent liver IFN signature and myeloid cells with increased IL-10 production after bacterial infection was also found in patients with liver cirrhosis. The augmented IFN and IL-10 expression incapacitated antibacterial immunity of myeloid cells and caused failure to control bacterial infection in severe liver fibrosis and cirrhosis (126–128). HSCs could also response to LPS by releasing pro-inflammatory cytokines (e.g., TNF-α, IL-6, and IL-8) and chemokines (e.g., CCL2, CCL5) and gained expression of adhesion molecules (55, 124). In addition to the TLRs, LPS can also activate inflammasomes by binding to NLRs, which leads to increased release of IL-1β and IL-18 (129, 130). These studies depicted changes in cytokine profile induced by PAMPs, providing potential therapeutic targets for liver diseases based on the gut-liver axis.

#### Recruitment of mucosal immune cells into the liver

In parallel to the ‘leaky gut’ as described above, the ‘gut lymphocyte homing’ is another supposed interaction between the gut and liver immune system. Primary sclerosing cholangitis is strongly linked to inflammatory bowel disease, in which the liver disease develops in the absence of a diseased colon. In this condition, some mucosal lymphocytes generate in the gut during active inflammatory disease and persist as long-lived memory cells are supposed to home to the liver (131). Subsequent studies showed that the CCR9^+^ gut-homing lymphocytes were recruited by gut-specific chemokine CCL25 expressed by the hepatic endothelium (76, 132). The LSECs also expressed increased levels of mucosal addressin cell adhesion molecule-1 (MAdCAM-1), inter-cellular adhesion molecule-1 (ICAM-1) and vascular-cell adhesion molecule-1 (VCAM-1) for lymphocyte adhesion (77, 132, 133). In addition, there were other studies revealed existence of T cells expressing clonally related TCRβ chain and recognizing the same antigen in the intestine and liver (134), and hepatic B cells that produce IgA deriving from intestinal lymphoid tissue (135). These phenomenon highlight the association of lymphocyte recruitment in gut-liver axis, and call for further exploration of other communication in this axis.

### Lung and hepatic immune regulation

Physiologically, the lung and liver are closely coordinated. When the liver function is perturbed, dysfunctional liver can lead to the abnormal expansion of pulmonary, triggering hypoxemia and a series of other pathophysiological changes and clinical symptoms known as hepato-pulmonary syndrome, which is common in patients with cirrhosis (78). Correspondingly, many hepatic manifests are often secondary to pulmonary disease such as pneumonia, due to the fact that mediators derived from the inflamed lungs can cause liver inflammation. Therefore, the pulmonary-mediated hepatic immune regulation will be reviewed in conditions of both lung disease and liver disease.

#### Hepatic acute phase response induced by the lung-liver axis

The acute-phase response (APR) is a prominent systemic reaction of the organism to local or systemic disturbances in its homeostasis, defined by significant changes in plasma concentrations of inflammation markers. These inflammation markers are acute-phase proteins (APPs). The liver is intensively involved in the APR of the organism in pneumonia and other cases (136). During pneumonia, inflammatory response caused within the airspaces is typified by cytokine production (e.g., IL-6, TNF, and IL-1), leukocyte recruitment and plasma extravasation (136, 137). Plasma extravasation could then induce the APR in the liver. The hepatocyte-derived APPs exert a direct role of curbing injury induced by TNF-α in the liver itself, but also promote survival in association with innate immunity in the lungs (136). The APR is an orchestrated response to tissue injury, infection or inflammation, and the APPs induced during this response act to limit proteolytic and/or fibrogenic activity and tissue damage, thereby contributing to the restoration of homeostasis (138, 139). APR provides novel signaling axis for the immune-mediated lung-liver communication (140, 141).

#### Pulmonary-derived inflammatory cytokines and hepatic immune regulation

Insult like chronic alcohol exposure results in both alcohol-related liver disease and alcohol-related susceptibility to acute lung injury. Alcohol-induced injuries to these two organs share a deal of parallel mechanisms, including: damages to both organs are involved to oxidative stress that favors tissue injury (142, 143), inflammatory injuries to both organs are enhanced by alcohol exposure (144, 145), and most importantly, dysregulated cytokine production in the development and progression of both diseases (146, 147). These phenomena indicate that there exists a “shuttle” between the two organs, promoting the pathogenesis of both organs. Study in the mechanically ventilated (MV) lung injury model provides evidence for this assumption: perfusate from injured lungs was able to cause a robust inflammatory response with significantly increased production of pro-inflammatory factors such as G-CSF, IL-6, CXCL1, CXCL2, and CCL2 in LSECs (148); liver tissues obtained from mice subjected to *in vivo* MV also demonstrated significant increases in hepatic gene transcription of IL-6, CXCL1, and CXCL2 (148).

TNF-α is a common mechanism of alcohol-induced pathology in both lung and liver (146, 149). In the lung, TNF-α led to elevated levels of TNF-α-responsive chemokines, CXCL2 and keratinocyte chemoattractant, all of which were correlated with increased pulmonary neutrophil recruitment (150). Moreover, in a chronic alcohol pre-exposure enhanced endotoxemia-induced acute lung injury model, the lung injury could be prevented by blocking systemic TNF-α with etanercept (147). In the liver, TNF-α activates several intracellular pathways to regulate inflammation, cell death, and proliferation, and is closely associated with liver injury (151). Therefore, although evidence about the predominate source of TNF-α is still lack, it is reasonable of us to speculate that TNF-α may act as one of the mediators that derived from the inflammatory lung to promote the occurrence of hepatic inflammation. Future studies are required to identify more mediators that contribute to the hepato-pulmonary association other than TNF-α.

### Adipose tissue and hepatic immune regulation

Alcoholic (ALD) and non-alcoholic fatty liver diseases (NAFLD) are clinical symptoms of hepatocellular injury and inflammation caused by alcohol consumption, high fat diet, obesity and diabetes, among others, and are both characterized by the expandability of adipose tissue. Anatomically, adipose tissue consists of visceral adipose tissue (VAT) and subcutaneous adipose tissue (152). VAT is mainly present within the abdominal cavity, and visceral fat venous blood is drained directly into the liver through the portal vein, and abnormal metabolic pathways and inflammation in VAT are implicated in the pathogenesis of ALD and NAFLD (153). Deregulated adipose tissue has increased lipolysis in adipocyte and activated inflammatory responses in adipose immune cells such as macrophages, which in turn lead to the release of free fatty acids, adipokines, and cytokines into the portal circulation  (154–157), and these factors are associated with hepatic immune regulation.

#### Adipose tissue-derived pro-inflammatory cytokines in hepatic immune regulation

Upregulated expression of pro-inflammatory cytokines (e.g., IL-6 and TNF-α) and chemokines (e.g., CCL2) in adipose tissue are observed in both alcoholic patients (158) and rodent models (159), particularly, the VAT is found to secret large quantities of IL-6 (160–162). The development of NAFLD and insulin resistance is also supposed to be resulted from imbalanced cytokines (increased pro-inflammatory and decreased anti-inflammatory cytokines) (163, 164). These pro-inflammatory cytokines can be delivered into the portal circulation, and directly associate with liver inflammation and fibrosis in hepatic steatosis.

#### Adipocyte-derived adipokines in hepatic immune regulation

Adipokines are a class of adipose-derived signaling molecules that contribute to the development of ALD and NAFLD. Adiponectin, one of the well-known adipokines, has insulin-sensitization and anti-inflammatory effects in insulin target tissues including liver, and acts as an important regulator for the development of hepatic diseases. Correlation between the onset of hepatic disease and reduced circulating adiponectin levels, decreased expression of adiponectin receptors, and impaired adiponectin-mediated signaling is shown in several animal models of hepatic syndromes (165). *Via* its cognate receptors, adiponectin receptors 1 and 2, adiponectin potently suppresses hepatic inflammation. KCs constitutively express AdipoR2 (166, 167), suggesting a role of KCs in adiponectin-mediated hepatic anti-inflammation properties. Adiponectin is also found has a role of blocking TNF-α-stimulated CCL2 expression, and thus resulting in reduced macrophage infiltration in the liver (168, 169). However, there is also data showing decreased adiponectin in the plasma of alcohol-fed rodents. This might due to increased TNF-α expression in adipose tissue caused by alcohol administration in rodents (170), and TNF-α could directly inhibit the release of adiponectin from the adipose tissue (171). Leptin, another important adipokine, is able to induce hepatic inflammation and fibrogenic responses by activating HSCs and KCs (172, 173). Increased production of leptin and decreased production of adiponectin were observed in alcoholic patients and mouse models (110). Therefore, different adipokines might have distinct roles in hepatic immune regulation, and their mechanisms might be complicated due to disease conditions.

#### Adipose tissue-derived EVs in hepatic immune regulation

Extracellular vesicles (EVs) are another important way by which adipose tissue transmits information to other organs, in addition to canonical hormones, growth factors and cytokines. EVs, including microvesicles (MVs) and exosomes or exosome-like vesicles (ELVs), are secreted by donor cells and transferred to the recipient cells, releasing encapsulated nucleic acids, lipids, and proteins to transfer information (134). Roles of adipose-derived exosomes in regulating liver metabolism have been widely documented both clinically and in animal models (174, 175), but their roles in modulating hepatic immune responses are less clear. Deng and colleagues (176) first found that adipose-derived exosomes of obese mice activated monocyte differentiation into adipose tissue macrophages (ATMs), leading to increased production of pro-inflammatory cytokines IL-6 and TNF-α. This process enhanced the migration of ATMs to liver and promoted the development of insulin resistance. ATMs accumulated in the liver also released miRNA-rich exosomes (e.g., miR-155) to regulate hepatic insulin sensitivity and inflammatory response (79). Exosomes from the adipose tissue derived mesenchymal stem cells were later demonstrated capable of promoting NK cells to exert antitumor roles on rat HCC, thereby inhibiting tumor growth (80). Together, these data indicate the possibility of adipose-derived EVs functioning as an intriguing mode for adipose tissues to regulate liver disease progression by modulating hepatic immunity.

### Brain and hepatic immune regulation

The brain and liver bidirectionally communicate *via* humoral and neural networks (177, 178). The neural axis between brain and liver interacts closely with the central nervous system (CNS) *via* the autonomic nervous system (ANS). The hepatic sympathetic and parasympathetic nervous systems are collectively known as the ANS, which is part of the peripheral nervous system and plays a key role in the regulation of numerous physiological events (including inflammation) in the liver (179, 180). The hepatic ANS transmits information from the liver to the CNS, and also receive signals from the CNS to regulate liver function, that is, the liver acts as both a sensor and effector affected by neurological signals.

The brain function can be severely affected in severe liver diseases with considerable inflammatory involvement, and these alterations in brain are associated with brain cholinergic dysfunction (181), which is involved with immune regulation. Cholinergic modulation of liver inflammation by the vagus nerve was first reported by Tracey and colleagues (182) more than 20 years ago. In the liver of rodents, they showed that electrical stimulation of the cervical vagus nerve could attenuate LPS-induced TNF production. In rats with hemorrhagic shock, Guarini and colleagues (183) demonstrated that the brain mAChR-mediated activation of efferent vagus nerve signaling to liver also caused significant suppression of hepatic TNF release. Later studies demonstrated a role of KCs in the cholinergic mediated modulation of hepatic immunity in several chronic liver diseases (182, 184, 185), indicating the involvement of immune cells in hepatic neuro-immune regulation. Such involvement was also demonstrated in hepatic NKT cells, which received signals from the catecholamine neurotransmitters, leading to phenotypic transformation (61, 186). Thus, neural signal-expressing cells involved with hepatic immune regulation deserve further study.

Pathogens are also triggers of the intrahepatic neuro-immune responses. On the one hand, immune cells in the liver could detect the presence of pathogen components and release cytokines (e.g., IL-1β and TNF-α) which function as chemical messengers. On the other hand, pathogens can also directly activate the hepatic neurons. These signals are transmitted and integrated in the CNS, and then descend *via* sympathetic and efferent vagus nerve fibers, releasing catecholamine and acetylcholine through hepatic nerve endings and modulating the liver immune response (181, 187). Particularly, in this brain-liver axis, hypothalamus is recognized to be the critical part for sensing and integrating signals from the periphery tissue and effecting appropriate changes to maintain metabolic and immunologic homeostasis (180, 188). Hypothalamic inflammation is shown an important event in brain-involved hepatic immune regulation and insulin resistance (189–191). As a summary, the nervous system and the immune system communicate in response to pathogen invasion, tissue injury, and other homeostatic threats. A systematic understanding of the mechanism by which dysregulated liver triggers hypothalamic inflammation is critical for realizing the nervous system mediated hepatic immune regulation.

### Other organs and hepatic immune regulation

There are also cross-talk between the liver and other organs, such as the BM, the pancreas, and the kidney, but the role of these organs in hepatic immune regulation remains to be further studied.

The BM is an immune-regulatory organ that has a role not only in hematopoiesis but also in immune responses (192). In addition to liver-resident immune cells, most inflammatory cells are derived from the BM (193, 194). MoMFs, key effector cells in the hepatic immune activities, are derived from infiltrated bone marrow-derived CCR2^+^CX3CR1^lo^Ly6C^hi^ monocytes, whose recruitment is dependent on the CCL2-CCR2 axis (195, 196), which has been well-recognized in plenty of liver diseases including liver fibrosis and hepatic carcinoma. The BM is also regarded as source of other leukocytes, however, a detailed depiction of how the BM is involved in hepatic immune regulation in other ways (e.g., cytokines, hormones, and exosomes) is still lacking.

Pancreas is a potential candidate extrahepatic organ to be involved in hepatic immune regulation. Fetuin-A secreted by the inflammatory liver could stimulate chemokines like CCL2 and IL-8, and pro-inflammatory cytokines such as IL-6 and IL-1β expression in the pancreas, and lead to damaged pancreas. In reverse, damaged pancreas may secrete pro-inflammatory cytokines (e.g., TNF-α) to directly attack the liver (197). Besides, there exists a gut-liver-kidney axis during the development and progression of chronic kidney disease associated with chronic fatty liver diseases. Kidney dysfunction led to metabolic acidosis, accumulation of toxins that have serious impacts on various liver functions, for example, changing glucose homeostasis, endothelial dysfunction, enhanced inflammation, and pro-inflammatory cytokines (198). Furthermore, the skin might also play a role in hepatic immune regulation. While clinical studies indicated that psoriasis may be more severe in patients with NAFLD/NASH (199, 200), livers from psoriatic mice were also found enriched for macrophages, polymorphonuclear neutrophils, and T cells (201).

In addition to the organs detailed in our review, correlation of other organs in hepatic immune regulation are also indicated, but the specific connection and exact mechanisms remain to be explored and validated. Nevertheless, the “shuttle” role of cytokines in these processes has been repeatedly mentioned, warranting our high attention to the overall changes of cytokines in the organism during disease development.

## Animal models and methods for studying the regulation of extrahepatic factors

### Animal models

To date, studies on the role of spleen in the regulation have provided the most evidence, but these evidence mainly come from splenectomy as an intervention. Novel animal models, including spleen-specific photo-conversion with KikGR transgenic mice (in which KikGreen cells are turned into KikRed by site-specific irradiation) (202, 203) and spleen transplantation (204) between congenic mice strain carrying differential markers, have been proven effective in studying the cell communication between spleen and other organs, employment of these models in studies about spleen-liver crosstalk may help reveal more details. As for other organs, some available models, such as bone marrow chimeras and CD11b-diphtheria toxin receptor mice, have been sophisticatedly used in studying recruitment of liver infiltrating macrophages from the peripheral (205, 206). Recently, Zhou et al. (207, 208) developed a multi-lineage tracing system for *in vivo* study of hematopoietic cell migration and development (basing on the Cre-loxP and Dre-rox dual recombinase), this could potentially be used to track the movement and differentiation of cells between organs.

### Methods for detection and tracking of inter-organ mediators

Generally, evidence that cytokines or exosomes from other organs influence hepatic immune response is not straightforward, because of the lack of ways to track these factors *in vivo*. The effects of these factors are always assessed by their corresponding changes in the target organs and liver, and the effect of these factors isolated from the target organs on liver cells during *in vitro* treatment. Real-time detecting, tracking and quantification of these factors will help assess their effects *in vivo*. Nanoparticle tracking analysis (NTA) is a technology developed based on the principle of light scattering and Brownian motion of particles in suspension and has been used for quantitative detection of exosomes (209). NTA also has different filters for analyzing fluorescent samples, so that exosomes with different markers on their surface (e.g., CD63, HSP70, and TSG-101) could be distinguished, and the results are more reliable than flow cytometry (210). For cytokine detection, various methods based on the antigen-antibody interaction (e.g., ELISA, ELISpot, bead-based flow assays) have been developed, but there are still many challenges. The impact and function of cytokines is directly linked to their extracellular expression levels, which often drastically vary with time and spatial location. Recent publications have suggested an interesting way forward for cytokine detection by combining molecular target-specific sensors that bind the respective analyte, and detection of successful binding through electric signals (211, 212). These biochips not only allow for fully automated detection of dozens to hundreds of cytokines in parallel, but also allow live and continuous detection of cytokines without the need to obtain any type of sample. However, methods for tracing and identifying the source of specific cytokine are not available yet.

## Conclusions and future perspectives

Liver disease is generally a multi-stage, multi-hit process, which may not only be the link between two organs, but also the link between several organs, especially in metabolic-related liver diseases such as ALD, NAFLD, and MAFLD. Hepatic immune alteration from homeostasis to activation is a complex process involving both intrahepatic and extrahepatic factors. The emerging understanding of cross-talk between the liver and other organs complements and completes our knowledge of the role of hepatic immune regulation in liver disease development. Better understanding of the origin specialization and cascade effects of shuttle mediators such as exosomes and cytokines like TNF-α, the trigger factors and recruitment mechanisms of immune cells from other organs to the liver, and the temporal and spatial changes of these events will provide the key to intervening in liver disease progression and other organ complications by modulating hepatic and systemic immunity. These findings will benefit the development of therapeutic strategies for liver diseases that target the cellular and molecular levels to minimize adverse reactions and maximize therapeutic effects. After all, there is an urgent need for more up-to-date models and methods relating to tracking and specific intervention to explore the role of extrahepatic factors in hepatic immune regulation.

## Author contributions

All authors listed have made a substantial, direct and intellectual contribution to the work, and approved it for publication.

## Funding

This work was funded by the National Natural Science Foundation of China (# 91842307 and 82173207 to ZL, # 82101915 to SZ), and the Second Affiliated Hospital of Xi'an Jiaotong University Foundation (# 2020YJ (ZYTS) 546-01 to SZ).

## Conflict of interest

The authors declare that the research was conducted in the absence of any commercial or financial relationships that could be construed as a potential conflict of interest.

## Publisher’s note

All claims expressed in this article are solely those of the authors and do not necessarily represent those of their affiliated organizations, or those of the publisher, the editors and the reviewers. Any product that may be evaluated in this article, or claim that may be made by its manufacturer, is not guaranteed or endorsed by the publisher.
